# Supplementary figures and images for: Anaerobiosis favors biosynthesis of single and multi-element nanostructures
Source: PLoS One. 2022 Oct 7;17(10):e0273392. doi: 10.1371/journal.pone.0273392 (PMC9543976; doi:10.1371/journal.pone.0273392)

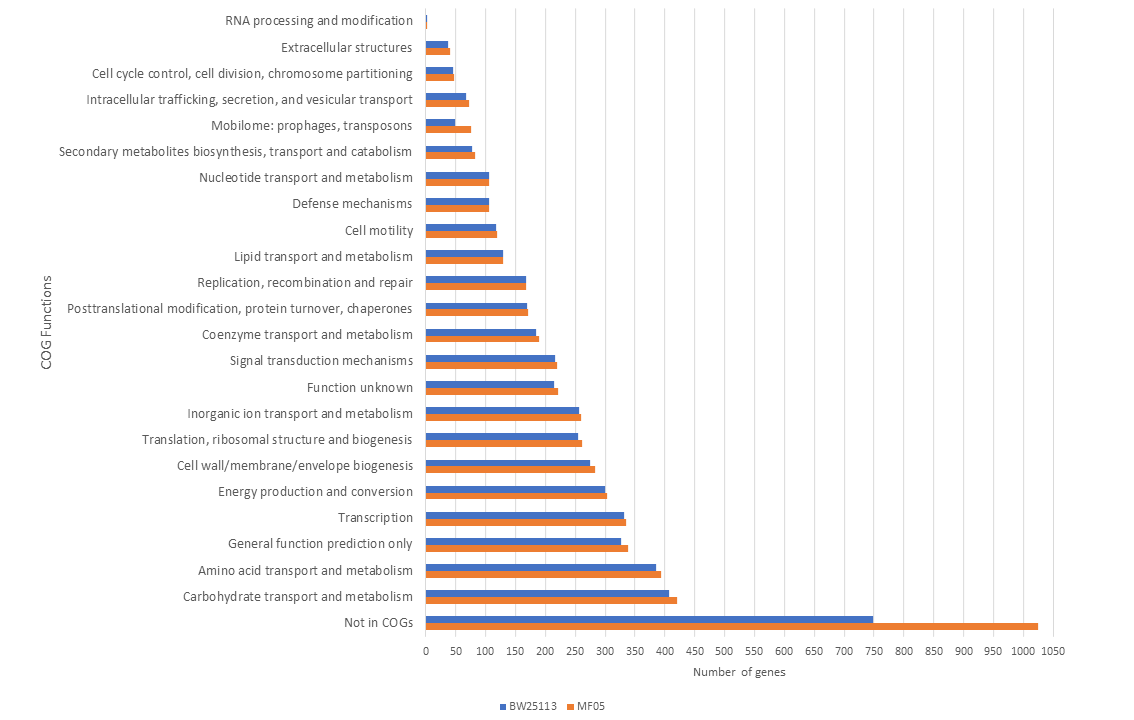

Supplement: S1 Fig — COG functions comparison for E. coli BW25113 (blue) and MF05 (orange). (TIF) [file pone.0273392.s001.tif]

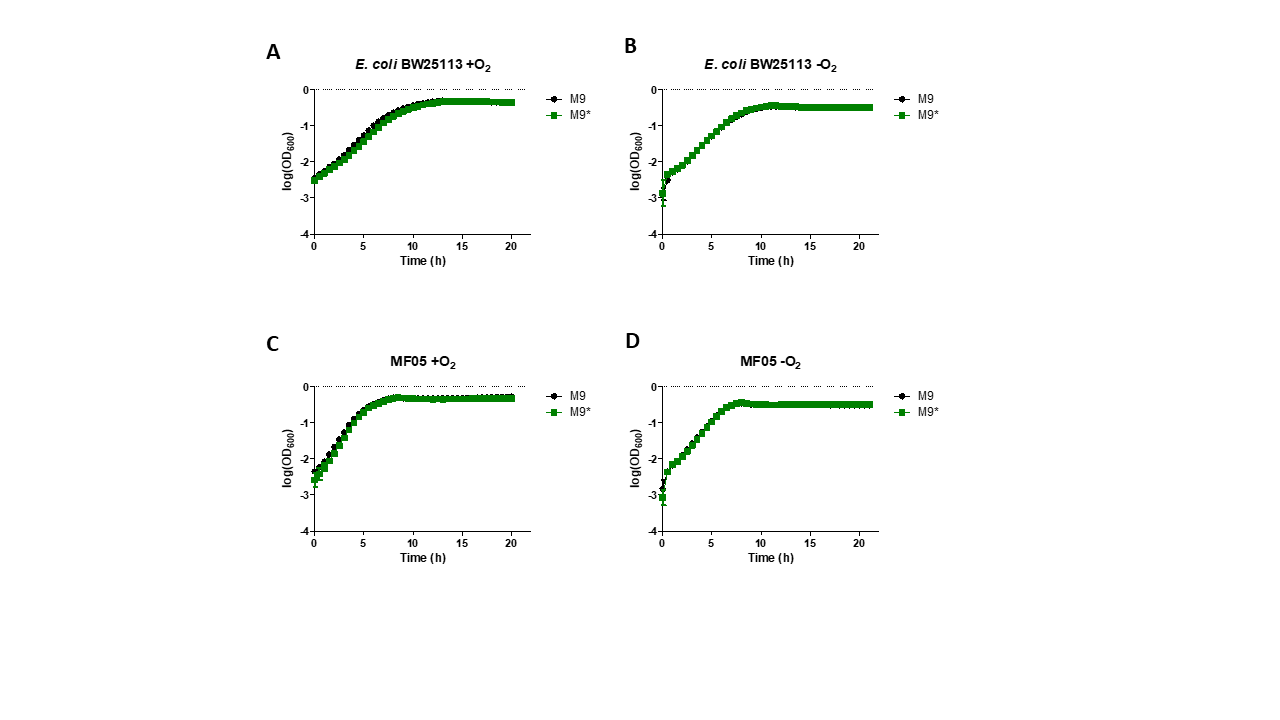

Supplement: S2 Fig — (A) Aerobic, (B) anaerobic E. coli BW25113, and (C) Aerobic, (D) MF05 cells grown in M9 (black circle) or M9* (inverted green triangle) medium. Each point represents the average of three independent trials ± SD. (TIF) [file pone.0273392.s002.tif]

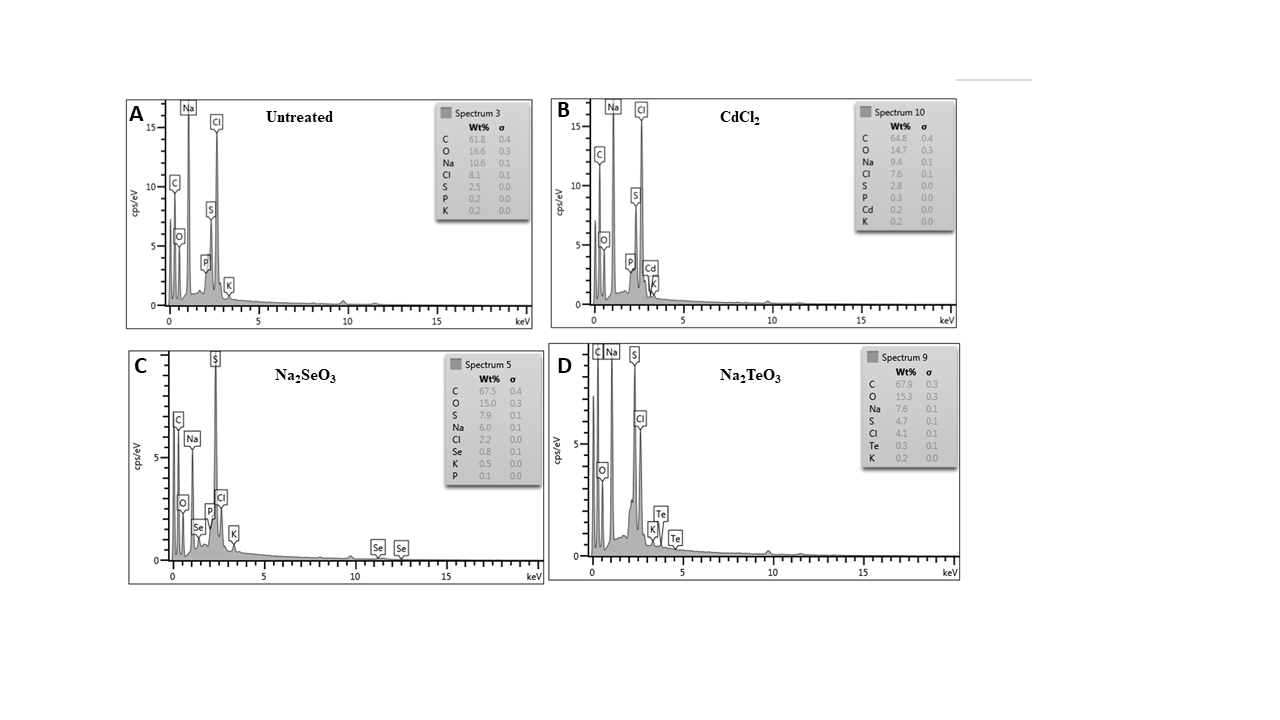

Supplement: S3 Fig — Whole cells were (A) left untreated, exposed to (B) only CdCl2, (C) only Na2SeO3 or (D) only Na2TeO3. (TIF) [file pone.0273392.s003.tif]

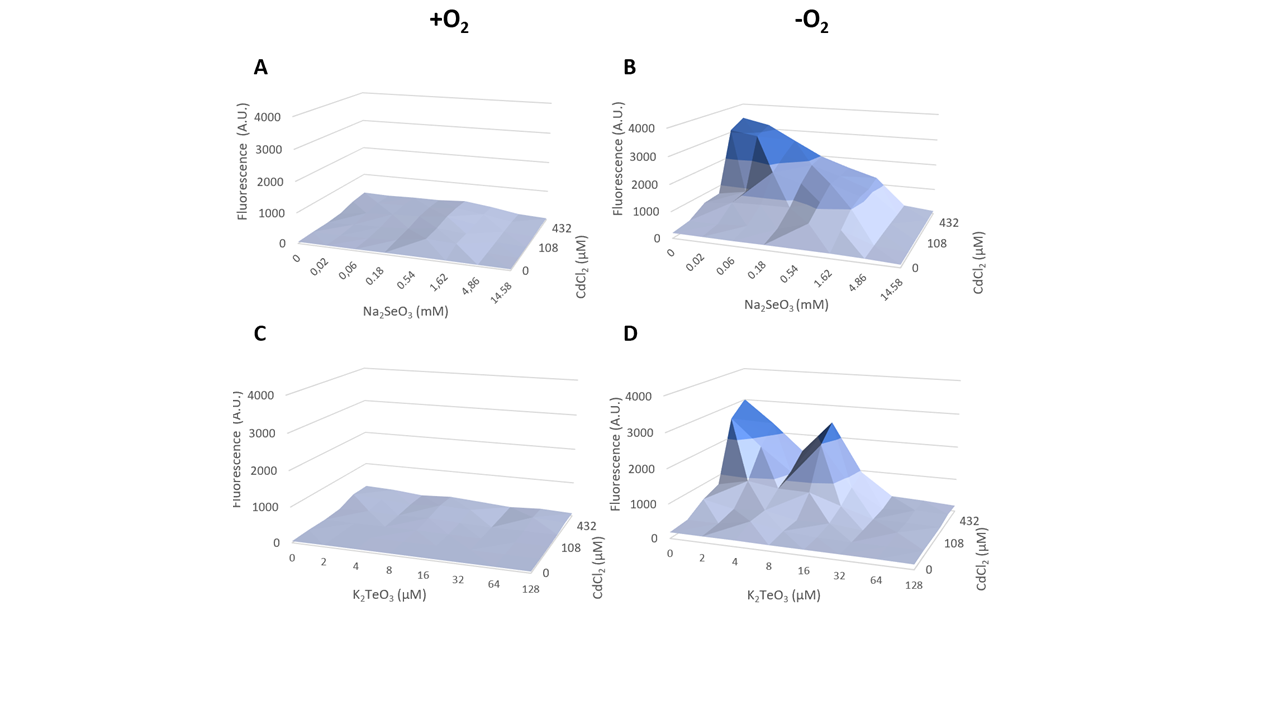

Supplement: S4 Fig — Fluorescence was monitored with 230 and 640 nm of excitation and emission wavelength, respectively. Na2SeO3 + CdCl2 in (A) aerobiosis or (B) anaerobiosis, and K2TeO3 + CdCl2 in (C) aerobiosis and (D) anaerobiosis. The results represent the average of three independent trials. (TIF) [file pone.0273392.s004.tif]

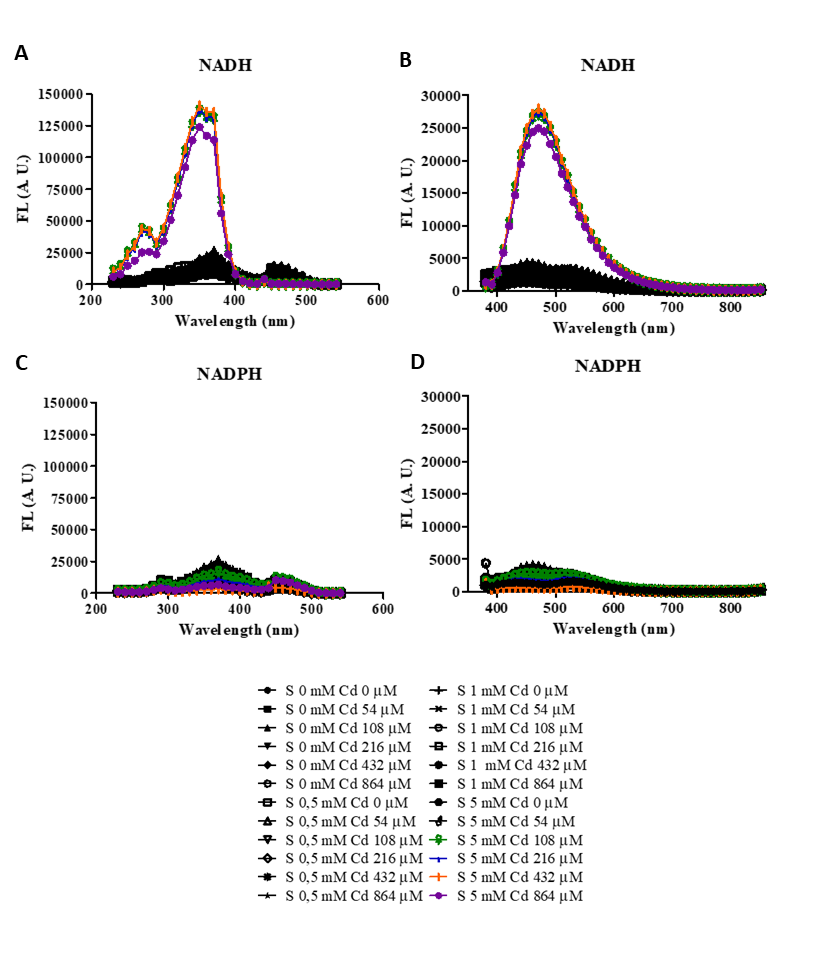

Supplement: S5 Fig — Excitation (A and C) and emission (B and D) scan spectra of crude extracts of MF05 treated with Na2SO3 (S) and/or CdCl2 (Cd) in the presence of NADH (A and B) or NADPH (C and D). (TIF) [file pone.0273392.s005.tif]

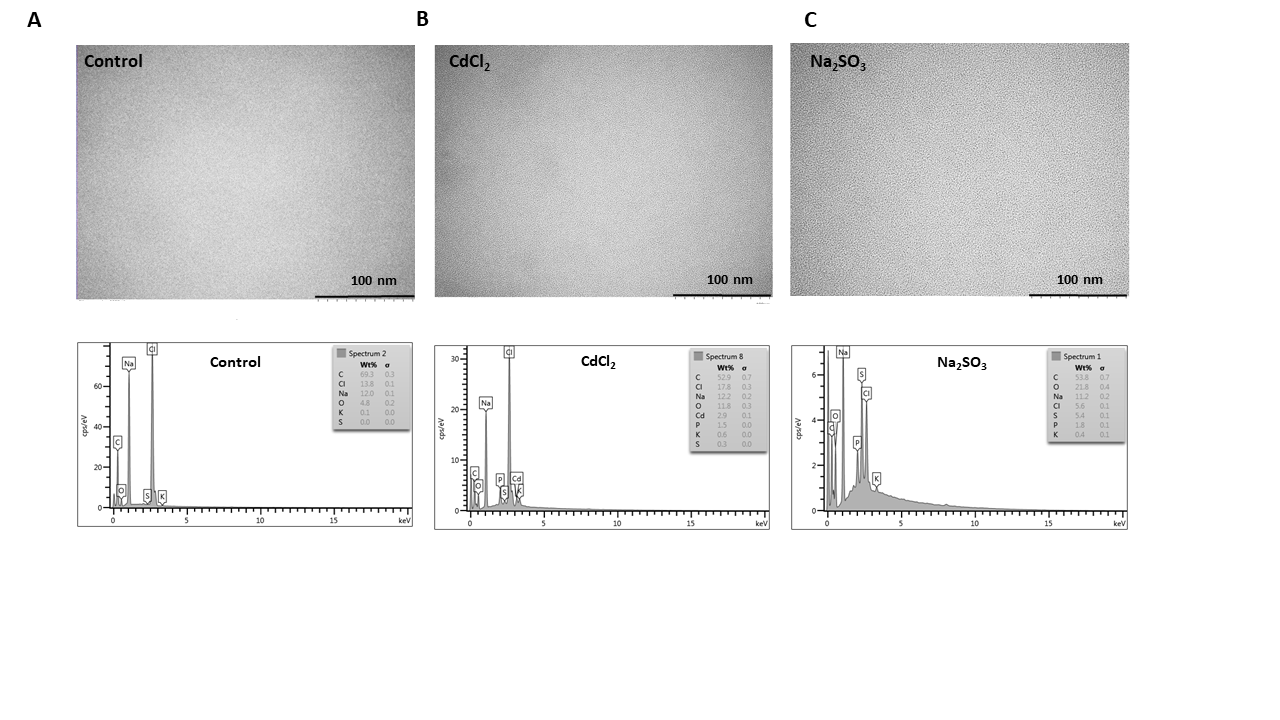

Supplement: S6 Fig — Whole cells were (A) left untreated, or exposed to (B) only CdCl2 or (C) only Na2SO3.TEM (upper images) and SEM EDX (lower images) showed no formation of NP under these conditions. (TIF) [file pone.0273392.s006.tif]

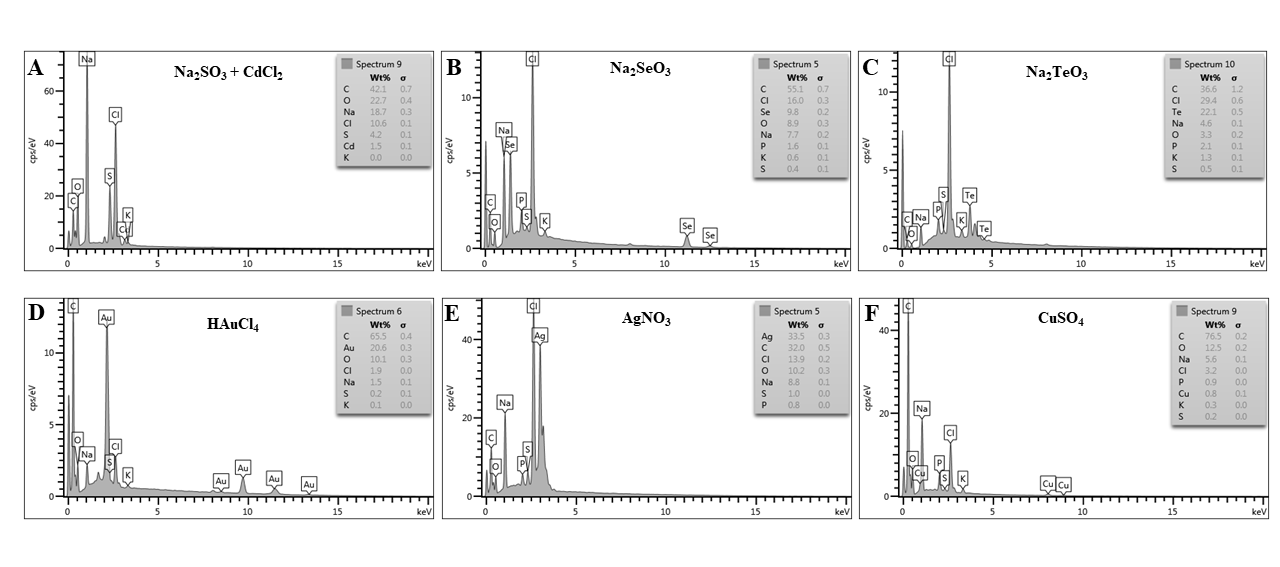

Supplement: S7 Fig — Cells extracts were treated with (A) Na2SO3 + CdCl2, (B)Na2SeO3, (C) Na2TeO3, (D) HAuCl4, (E) AgNO3 or (F) CuSO4. (TIF) [file pone.0273392.s007.tif]
