# Supplementary material for: Anaerobiosis favors biosynthesis of single and multi-element nanostructures
Source: PLoS One. 2022 Oct 7;17(10):e0273392. doi: 10.1371/journal.pone.0273392 (PMC9543976; doi:10.1371/journal.pone.0273392)
Supplement: Attachment — Submitted filename: Rebuttal Letter ENE2022.docx [file pone.0273392.s008.docx]

**February 23, 2022**

**Rebuttal Letter**

**Dear Plos One Journal team:**

Thank you for your feedback in our work. According to the notification received, we modified our manuscript, and all the changes made were highlighted in a document. We complemented the whole work with the queries and comments from the journal, editor and reviewer described below:

**Journal requirements:**

**Query 1:** Please ensure that your manuscript meets PLOS ONE's style requirements, including those for file naming.

**Answer 1:** We checked all the files and format to comply with the requested format, including file naming.

**Query 2:** We note that the grant information you provided in the ‘Funding Information’ and ‘Financial Disclosure’ sections do not match.

**Answer 2:** We completed the funding information with the following paragraph:

This work received financial support from FONDECYT (Fondo Nacional de Ciencia y Tecnología) Regular 1160051 (CV), National doctoral scholarship CONICYT (Comisión Nacional de Investigación Científica y Tecnológica) 21170508 (MR), support from USA1799 Vridei (Vicerrectoría de Investigación, Desarrollo e Innovación) 021943CV_GO Universidad de Santiago de Chile (MR, CV), Basal FB0807 CEDENNA (EV) and Centro de Genómica y Bioinformática, Universidad Mayor (JV) is also acknowledged.

**Query 3:** We note that you have stated that you will provide repository information for your data at acceptance. Should your manuscript be accepted for publication, we will hold it until you provide the relevant accession numbers or DOIs necessary to access your data. If you wish to make changes to your Data Availability statement, please describe these changes in your cover letter and we will update your Data Availability statement to reflect the information you provide.

**Answer 3:** We deeply apologize for this misunderstanding since I thought you were referred to the sequence data. We added to the cover letter that we don’t have the data in a repository information. We wish to change the Data Availability Statement.

**Query 4:** We note that you are reporting an analysis of a microarray, next-generation sequencing, or deep sequencing data set. PLOS requires that authors comply with field-specific standards for preparation, recording, and deposition of data in repositories appropriate to their field. Please upload these data to a stable, public repository (such as ArrayExpress, Gene Expression Omnibus (GEO), DNA Data Bank of Japan (DDBJ), NCBI GenBank, NCBI Sequence Read Archive, or EMBL Nucleotide Sequence Database (ENA)). In your revised cover letter, please provide the relevant accession numbers that may be used to access these data. For a full list of recommended repositories, see <http://journals.plos.org/plosone/s/data-availability#loc-omics> or <http://journals.plos.org/plosone/s/data-availability#loc-sequencing>.

**Answer 4:** We clarified the preparation method in Materials and Methods section, lines 140-144 and we uploaded the sequence to NCBI GenBank, with Reference Sequence: NZ_VCSI00000000.1. You can go directly through the following link, in which all contigs are available with their respective sequences:

<https://www.ncbi.nlm.nih.gov/nuccore/1679187305>

And also in here: <https://www.ncbi.nlm.nih.gov/Traces/wgs/VCSI01?display=contigs&page=1>

**Query 5**: Please review your reference list to ensure that it is complete and correct. If you have cited papers that have been retracted, please include the rationale for doing so in the manuscript text or remove these references and replace them with relevant current references. Any changes to the reference list should be mentioned in the rebuttal letter that accompanies your revised manuscript. If you need to cite a retracted article, indicate the article’s retracted status in the References list and also include a citation and full reference for the retraction notice.

**Answer 5:** All References were checked and corrected according to the format requested and the number 33 was recently added to the discussion section, suggested by the reviewer.

**Additional editor comments**

**Query 1:** The reviewer identified some shortcomings. Please specifically see to the comment on animal vs. bacterial models.
**Answer 1:** All the comments were kindly incorporated to the manuscript. We changed all the “in-vivo” terms for “whole-cell”.

**Reviewers' comments**

**Query 1:** what about the cytotoxic effect of using Bacteria in your experimental study?

**Answer 1**: Previous works found virulence genes in several *E. coli* environmental isolates. Specifically, *stx1*(Shiga toxin 1), *stx2* (Shiga toxin 2), *eaeA* (Attaching and effacing protein) and *hlyA* (hemolysin). Then, we examined our genome data and found two hemolysin genes in MF05 strain. This point was discussed in the manuscript in lines 380-390, and the reference was added.

**Query 2:** What is the stability of the particle size of bacteria used for the study?

**Answer 2:** This is a very important point, and during our experimental procedures, we were able to see the stability of the particles size, since fluorescence properties of QDs were present for at least 10 weeks at 4° C. Fluorescence in QDs is directly related to their size, so it might be possible that the bioprocess itself confers biostabilizers that could be very interesting to characterize in future experiments. This point was added in the discussion in lines 352-356

**Query 3:** Since the author has concluded the materials can be used for industrial applications, in this regard if the particle is going to be used in pharmacy or drug preparation, what is rating of its biocompatibility.

**Answer 3:** This comment was discussed inside our group and is absolutely necessary to characterize biocompatibility to define in a specific way, which uses can have these nanostructures. A comment related to this point was added in the discussion section in lines 379-381.

**Query 4:** The *in vivo* studies is always in animal model not in bacterial samples. Kindly correct in the manuscript and update the datas.

**Answer 4:** We changed all the “*in-vivo*” synthesis terms for “whole-cell” synthesis, claryfing that these are bacterial samples in which cell cultures are used to synthesize nanostructures.

We appreciated the feedback, and we hope that our answers are satisfactory for all of you.

Sincerely,

Dr. Felipe Arenas Salinas

Associated Profesor
Universidad de Santiago de Chile
