# Supplementary material for: Anaerobiosis favors biosynthesis of single and multi-element nanostructures
Source: PLoS One. 2022 Oct 7;17(10):e0273392. doi: 10.1371/journal.pone.0273392 (PMC9543976; doi:10.1371/journal.pone.0273392)
Supplement: Attachment — Submitted filename: Respuesta referee PAPER NS anaerobico MR.docx [file pone.0273392.s009.docx]

July 29, 2022

**Rebuttal Letter**

**Dear Plos One Journal team:**

Thank you for your feedback in our work. According to the notification received, we modified our manuscript, and all the changes made were highlighted in a document. We complemented the whole work with the queries and comments from the journal, editor and reviewer described below:

**Requirements:**

1. Abstract is so weak and have to support with some data.

-The abstract has been rewritten and updated as requested.

2. Authors neglect big survey for recent works for inorganic mono and bimetallic nanoparticles and their applications. These recent publications are quite important to cite [Biocatalysis and Agricultural Biotechnology 2022, 39, 102261; Surfaces and Interfaces 2021, 25, 101175; Carbohydrate Polymers 2021, 266, 118163; Journal of Molecular Liquids 2021, 321, 114669; Polymer Testing 2020, 89, 106720; Rsc Advances 2016, 6 (78), 73974-73985; International journal of biological macromolecules 2019, 138, 450-461; International Journal of Biological Macromolecules 2020, 156, 829-840].

-We update the reference section. We apologize since, this paper was submitted last year, peer-reviewed in February and now re-peer-reviewed. The publications recommended have been include as references.

3. Number of references in the last five years is less than 30% and this is not sufficient. Number of recent works has to increase for double at least.

 -We updated the reference list, with 20 works in the last five years.

4. Number of literatures has to increase. 33 references are quite low numbers.

-We updated and increase the reference list, now there are 56 references.

5. Improve the quality of figures, as they are not clear enough. High resolution figures are required.

 -We improved the resolution of the figures.

6. All TEM micrographs have to include the same bare size. Check Figures 4, 6,

- It results us difficult to have the same bar size, since different precursors for nanostructure synthesis have been used, so the biological components related to synthesis might be different so as the sizes obtained. In addition, TEM micrographs were an external service so given the complexity related to all processes needed to recharacterize synthesis products, we could say we would not be able to obtain new TEM images.

7. Size distribution of the synthesized nanoparticles has to measure.

-We made measurements by DLS, however the polydispersity index was very high, given that the biological method has many impurities. In addition, the nanostructures obtained are of an irregular type.

8. FTIR is required for the synthesized nanoparticles.

- We didn’t consider IR characterizations because these nanoparticles, obtained through biological processes using bacteria, were not purified so there might be a lot of organic components attached that would interfere roughly in this kind of characterization.

9. Compare the size of nanoparticles with literature [Surfaces and Interfaces 2021, 25, 101175; Carbohydrate Polymers 2021, 266, 118163; Journal of Molecular Liquids 2021, 321, 114669; Polymer Testing 2020, 89, 106720].

-Unfortunately, and given that the biological synthesis of nanoparticles is not a process directly comparable to chemical synthesis, mainly due to the presence of biological material (proteins or metabolites) which interfere with the calculation of size and direct comparison with nanoparticles or marked manuscripts.

10. Compare the fluorescence of quantum dots with CQDs in literature [RSC Advances 2020, 10 (70), 42916-42929; International Journal of Biological Macromolecules 2021, 170, 688-700; Journal of Colloid and Interface Science 2021, 604, 15-29; Cellulose 2021, 28 (15), 9991-10011].

-The spirit of this paper is not to compare fluorescence properties between different QDs, but to study reduction capacity of bacteria under anaerobiosis to produce nanostructures. Fluorescence properties of CdS were used as an indicator of nanostructures synthesis. However, in the future quantum yields and other studies might be conducted in order to compare QDs from different natures (carbon and chalcogenides).

11. Conclusion is so short and have to support with more information.

-The conclusion has been deleted, according to the Journal guidelines it is optional to be written.

12. Check and revise the language in whole manuscript.

-The whole manuscript has been checked and revised, in both grammar and orthography.
